# The Effect of Salinity Stress on Enzyme Activities, Histology, and Transcriptome of Silver Carp (*Hypophthalmichthys molitrix*)

**DOI:** 10.3390/biology11111580

**Published:** 2022-10-27

**Authors:** Yuhan Jiang, Chen Yuan, Ming Qi, Qigen Liu, Zhongjun Hu

**Affiliations:** 1 Centre for Research on Environmental Ecology and Fish Nutrition of the Ministry of Agriculture, Shanghai Ocean University, Shanghai 200120, China; 2 Key Laboratory of Exploration and Utilization of Aquatic Genetic Resources, Ministry of Education, Shanghai Ocean University, Shanghai 200120, China; 3 Shanghai Engineering Research Center of Aquaculture, Shanghai Ocean University, Shanghai 200120, China; 4 Zhejiang Fisheries Technical Extension Center, Hangzhou 310000, China

**Keywords:** silver carp, *Hypophthalmichthys molitrix*, salinity, enzyme activities, histology, transcriptome

## Abstract

**Simple Summary:**

Salinity is a key environmental driver for many aquatic animals and can regulate biological characteristics such as growth, reproductive behavior, and survival. Silver carp (*Hypophthalmichthys molitrix*) is an economically and ecologically important fish species in China. In the current study, we compared juvenile silver carp in freshwater versus brackish water (6‰ salinity) to determine differences in antioxidant enzyme vitality index activity (SOD, CAT, GSH-PX), Na+/K+-ATPase (NKA), histology, and differentially expressed genes (DEGs) toward achieving comprehensive understanding of the physiology, ecological habits, and salinity tolerance mechanisms of this species. A theoretical basis for the establishment of the brackish water aquaculture in saline–alkali land may be framed by advancing and perfecting the fundamental biological research in silver carp.

**Abstract:**

A 56-day study was performed to examine the effect of freshwater (FW) and brackish water (BW 6‰ salinity) on the antioxidant ability, Na+/K+-ATPase (NKA) activities, histology, and transcriptome of the gill and kidney tissue in juvenile silver carp (*Hypophthalmichthys molitrix*). The results show that when juvenile silver carp were exposed to 6‰ salinity, the activities of superoxide dismutase (SOD) and catalase (CAT) were shown to be substantially increased (*p* < 0.05), while glutathione peroxidase (GSH-PX) activities in gill were not significantly affected (*p* < 0.05). In kidney tissue, SOD, CAT, and GSH-PX, enzyme activities peaked at 24, 8, and 4 h, respectively, but were not significantly different compared with the control group (*p* < 0.05). In addition, significant effects of salinity were observed for the NKA level in both the gills and kidney tissues (*p* < 0.05). The gill filaments of juvenile silver carp under the BW group all underwent adverse changes within 72 h, such as cracks and ruptures in the main part of the gill filaments, bending of the gill lamellae and enlargement of the gaps, and an increase in the number of mucus and chloride-secreting cells. Transcriptome sequencing showed 171 and 261 genes in the gill and kidney tissues of juvenile silver carp compared to the BW group, respectively. Based on their gene ontology annotations, transcripts were sorted into four functional gene groups, each of which may play a role in salt tolerance. Systems involved in these processes include metabolism, signal transduction, immunoinflammatory response, and ion transport. The above findings indicate that the regulation processes in juvenile silver carp under brackish water conditions are complex and multifaceted. These processes and mechanisms shed light on the regulatory mechanism of silver carp osmolarity and provide a theoretical foundation for future research into silver carp growth in brackish water aquaculture area.

## 1. Introduction

The influence of salinity on fish is a complex physiological process. Even though some freshwater fish have been cultivated in saline–alkali water with some degree of success, the survival rates of these fish are significantly impacted by the varying levels of salinity and alkalinity in their environments. Studies have shown that high salinity will have a certain impact on the growth, reproduction, and metabolism of freshwater fish [1,2]. In the actual process of artificial culture, the appropriate water salinity and reasonable salinity regulation mode can not only reduce the outbreak of freshwater fish diseases but can also have important economic and scientific significance for the low-salinity domestication of freshwater fish, as well as the cultivation of low-lying saline–alkali land brackish water and artificial breeding.

Salinity stress can disrupt biochemical homeostasis in the cell membrane, resulting in the production of a large number of reactive oxygen species (ROS) [3,4]. A disruption in the homeostasis between reactive oxygen species (ROS) and antioxidant capacity leads to oxidative stress, which adversely affects animals [5]. The primary defense of cells from oxidative stress is provided by antioxidant enzymes, in which superoxide dismutase (SOD), glutathione peroxide (GSH-PX), and catalase (CAT) enzyme cascades represent the first line of defense against ROS [6,7]. Fish have systems of antioxidant enzymes, including SOD, GSH-PX, and CAT [8], that play an important role in scavenging excessive reactive oxygen radicals, alleviating self-damage, and improving the defense ability of immune cells [9]. Under unsuitable salinity environments, the antioxidant enzyme system is an important immune defense system for the fish body against environmental stresses [10]. Na+/K+-ATPase (NKA) enzymes are closely linked with osmolarity regulation, and the in vivo maintenance of osmolarity balance involves active transmembrane transport of Na+ and K+ ions and, thus, can be used as a major measure of osmolarity regulation in fish [11]. Osmoregulatory epithelia, such as fish gills, rely on osmolarity regulation for the maintenance of intracellular homeostasis and to power a number of transportation systems [12].

Changes in salinity alter the gill microstructure, resulting in the production and recruitment of mitochondria-rich cells (MRCs) in the interlamellar cell mass (ILCM) of European sea bass (*Dicentrarchus labrax*), Arctic grayling (*Thymallus arcticus*), and rainbow trout (*Oncorhynchus mykiss*) [13,14,15,16]. In addition to morphological plasticity, salinity perturbation alters fish physiological status, and the changes in plasma/serum ion concentrations and osmotic pressure coincide with changes in metabolic rates and gene expression patterns [17,18,19]. Anadromous fish that migrate often have high osmotic plasticity, which allows them to survive in a wide range of salinities. For example, when chum salmon migrate to saltwater, the expression of ion binding, transmembrane transporter, ion transport, and water channel genes was shown to be higher, revealing an osmoregulation network in which there are interactions with one another [20]. These observations suggest that osmoregulation in fish requires the coordination of molecular, cellular, and physiological mechanisms [21].

Silver carp (*Hypophthalmichthys molitrix*) is one of the four major domestic fishes farmed in freshwater in China and is an important freshwater fish in breeding. Because inland waters are more polluted and cyanobacteria blooms frequently occur, silver carp are often exiled into lakes and reservoirs, where they proliferate extensively, to control the abnormal growth of algae and obtain economic benefits [22,23]. Currently, many studies have been conducted on the effects of salinity on freshwater fish [24,25,26]. Some freshwater fish have been successfully cultivated in saline–alkali water, including crucian carp (*Carassius auratus gibelio*), grass carp (*Ctenopharyngodon idella*), bighead carp (*Hypophthalmichthys nobilis*), medaka (*Oryzias latipes*), and channel catfish (*Ictalurus punctatus*) [26]. This not only results in the improved utilization rate of saline–alkali water but is also important economically for the aquaculture of freshwater fish in saline–alkali areas. In 2012, China also released water quality standards for aquaculture on saline–alkali land. The saline–alkali water quality of class I was suitable for freshwater fish [27]. In this study, we examine the differences in the enzyme vitality index values, histology, and transcriptomics of juvenile silver carp in BW (6‰ salinity) toward developing a more comprehensive understanding of its physiological and ecological habits and salt tolerance mechanisms. In order to establish a brackish water aquaculture area in the resource-rich saline–alkali land zone, it is helpful to refine and enhance the basic biological research on silver carp.

## 2. Materials and Methods

### 2.1. Fish Preparation and Maintenance

Healthy juvenile silver carp were transferred from Huzhou Ecological Agriculture Technology Co, Ltd. (Huzhou, Zhejiang Province, China) to the laboratory of Ecology in Aquaculture and Fisheries of Shanghai Ocean University (Shanghai, China). Healthy individuals (102 days old, 104.69 ± 3.08 g weight, and 14.65 ± 0.46 cm total length) were acclimated in plastic culture tanks (1.2 m × 1 m × 1 m, water volume: 1200 L) for 15 d. The water temperature was maintained at 23.2 ± 2.0 °C, dissolved oxygen at 6.56 ± 0.20 mg/L, and salinity at 0 ppt, and the light cycle was set as the natural light cycle(laboratory natural light).During the period of acclimation, fish received artificial feed (30% crude protein content, 3% crude fat content; Techbank, Shanghai, China) according to 1% of their body weight twice daily (07:00 a.m., 6:00 p.m.). Cleaning was performed once a day at 8:00, and the water was changed once at 18:00 (50% of the volume in the tank).

### 2.2. Experimental Design and Sample Collection

After 15 days of acclimation, 180 healthy fish with strong individual vitality were used for salinity stress experiments and then placed into either of two groups (each with three replicates). According to the previous results, the silver carp died in a large scale at 7‰ salinity and were stressed at 6‰ salinity, therefore we used 0‰ (freshwater control group, FW) and 6‰ (brackish water group, BW) salinity for the groups. Aerated tap water was used in the experimental group to dilute seawater with 33% salinity, whereas directly aerated tap water was used in the control group. There were three replications for each treatment (30 fish per tank). The experiment lasted for 56 days, and fish were hand-fed to apparent satiation twice daily (07:00 and 18:00) with artificial feed (30% crude protein, 3% crude fat). Every day at 8:00, cleaning was performed, and every day at 18:00, water was changed to fill the tank to 50% of its capacity.

At the time points of 0, 2 h, 4 h, 8 h, 12 h, 24 h, 48 h, 72 h, 96 h, 15 d, 30 d, and 56 d, 6 fish were randomly selected from each fish tank and euthanized using 0.3 mg/L MS-222. The gill and kidney tissues were quickly frozen in liquid nitrogen and stored in the refrigerator at −80 °C, and the antioxidant capacity and NKA enzyme activity were then measured. Subsequently, 6 silver carp were randomly taken from each group and euthanized using 0.3 mg/L MS-222 and their gill tissues were dissected and stored in Bouin’s solution until being used for HE staining and histological observation. Finally, 3 juvenile silver carp were randomly picked from each of the tanks, for a total of 6 fish, and euthanized using 0.3 mg/L MS-222. Their gills and kidneys were removed and frozen immediately in liquid nitrogen. The samples were stored in a freezer at −80 °C for RNA-seq analysis.

### 2.3. Assay of Enzyme Activities

A ratio of 1:9 (weight/volume) of tissue to ice-cold physiological saline solution was used to separately homogenize both the kidneys and the gills. The supernatants were separated and stored at 4 °C after centrifugation (5000× *g* for 10 min at 4 °C). The Bradford method was used to determine the protein concentration of the enzyme extracts [28]. Superoxide dismutase (SOD) activity was measured using an SOD activity colorimetric assay kit (catalogue number: A001-1, Nanjing Jiancheng Bioengineering Institute, Nanjing, China). In the assay, SOD was combined with free radicals, and formed nitrite. The formed nitrite can react with a chromogenic agent and generate a purple color that can be read at k = 550 nm on a spectrophotometer. Catalase (CAT) activity was measured using a CAT activity colorimetric assay kit (catalogue number: A007-1, Nanjing Jiancheng Bioengineering Institute, Nanjing, China). In the assay, the reaction of CAT cleavage of substrates could be stopped with ammonium molybdate, and the remaining substrates reacted with ammonium molybdate by releasing chromophore, which was measured at 405 nm. The activity of sodium–potassium adenosine triphosphatase (Na+/K+-ATPase) was measured using a Na+/K+-ATPase activity colorimetric assay kit (catalogue number: A070-2, Nanjing Jiancheng Bioengineering Institute, Nanjing, China). In the assay, Na+/K+-ATPase cleaves ATP into ADP and inorganic phosphorus, and the amount of inorganic phosphorus was measured at 636 nm. Glutathione peroxidase (GSH-PX) activity was qualified using a GSH-PX colorimetric assay kit (Catalogue No. A005, Nanjing Jiancheng Bioengineering Institute, Nanjing, China). Specific enzymatic activities were expressed as the units per milligram of protein (U mg^−1^ protein). Soluble protein of crude enzyme extracts was quantified using the bicinchoninic acid protein assay kit (Cata-logue No. BCA1; Sigma-Aldrich, St. Louis, MO, USA).

### 2.4. Histological Examination Structure

Tissue samples from the gill filaments of juvenile silver carp were removed from the fixative and embedded in a paraffin section. Before sectioning and embedding in paraffin, gill samples were dehydrated using a series of graded ethanol solutions. Hematoxylin solution was applied for 3–5 min and washed off with distilled water, and hematoxylin differentiation solution was then applied, followed by hematoxylin Scott tap bluing, and the samples were finally rinsed. After being dyed with eosin dye for 5 min, slices were dehydrated in a series of solutions of ascending ethanol concentration, with 70% ethanol for 5 min, 85% ethanol for 5 min, 95% ethanol for 5 min, and absolute ethanol for 5 min in succession. Following this, images of the samples were taken using a light microscope (100×, 200×, 400×).

### 2.5. RNA-Seq

#### 2.5.1. RNA Extraction, Library Preparation, and Sequencing

Total RNA was extracted and purified from gills and kidneys (*n* = 3 per group) using TRIzol reagent (Invitrogen, Carlsbad, CA, USA) according to the manufacturer’s protocol and according to gill of the freshwater group (FWG), gill of the brackish water group (BWG), kidney of the freshwater group (FWK), and kidney of the brackish water group (BWK). NanoDrop 2000 (NanoDrop, Wilmington, DE, USA) was used to determine RNA quantity and purity for each sample. A sequencing library was constructed using only high-quality RNA samples (OD 260/280 ≥ 1.9, OD 260/230 ≥ 1.5, RIN ≥ 8.0). Major Bioinformatics Technology Co., Ltd., Shanghai, China, reverse-transcribed these RNAs into cDNA for sequencing as 150 bp paired-end reads on an Illumina NovaSeq 6000 (Illumina, San Diego, CA, USA) (Shanghai, China).

#### 2.5.2. Quality Control and Sequence Alignment

After CASAVA base calling, the Illumina Novaseq 6000 sequencing system converted the image signals of sequences into text data, which was stored in FASTQ format as raw data (raw reads). Using SeqPrep and Sickle software [29] to remove splice and N (N: modular base) sequences from raw reads and filter low-quality sequences (quality value < 20), clean reads were obtained following quality control. Following quality control, the error rate, Q20, Q30, and GC contents of clean reads were calculated to assess sequencing quality. Clean reads were mapped to the silver carp genome (*Hypophthalmichthys molitrix* CNA001988) based on the improved BWT (Burrows–Wheeler transform) algorithm and HISAT software version 2-2.1.0 (Daehwan, K Core Team, Austin, TX, USA) [30].

#### 2.5.3. Transcriptome Assembly and Annotation

Using the genome information of silver carp as a reference, the mapped reads were spliced and assembled into transcripts using Cufflinks software version 2.2.1 (Cole, T Core Team, Cambridge, MA, USA) [31] software. Unknown genes and novel transcripts were found by using GffCompare to compare the assembled transcripts to previously known transcripts. Annotation of the assembled transcriptomes was performed using BLAST + v.2.9.0 in conjunction with the NCBI non-redundant protein (Nr represent as follows), Swiss-Prot (Swiss-Prot), Clusters of Orthologous Groups of proteins (COG), GO Ontology (GO), Kyoto Encyclopedia of Genes and Genomes (KEGG), and Pfam protein family (Pfam) databases.

#### 2.5.4. Differential Expression Analysis of Genes and Enrichment Analysis

The TPM (transcripts per million reads) value in RESM v1.3.1 software was used to calculate gene expression values. Using DEGseq2 v. 1.24.0 [32] and one scaling normalization factor, the read counts of libraries from two experimental groups were modified. During the statistical calculation of differential expressions, the Wilcoxon rank sum test was used to adjust *p*-values to obtain adjusted *p*-values (*P*-adjust). The threshold for significant differences was set at *P*-adjust < 0.05 and |log2(foldchange)| ≥ 1 to identify differentially expressed genes. The software Goatools v. 0.6.5 [33] was utilized for GO enrichment analysis of DEGs in order to identify the DEGs’ primary GO functions. Using KOBASv.2.1 [34], the DEGs were analyzed, and KEGG enrichment results were obtained. GO functions and KEGG pathways were deemed significantly enriched when *P*-adjust < 0.05.

### 2.6. Real-Time Quantitative PCR Validation

Quantitative real-time PCR (qRT-PCR) analysis of 10 differentially expressed genes was performed to validate the transcriptome sequencing results. β-Actin was used as a reference gene. Primers for qRT-PCR were designed using Primer Premier v.5.0 and details are listed in Supplemental. The quantitative qRT-PCR analysis was conducted using the Prism 7500 Sequence Detection 140 System (Applied Biosystems, Shanghai, China) with a 2X SG Fast qPCR Master Mix (Sangon Biotech, Shanghai, China) for each sample, and qRT-PCR analysis was performed on three biological replicates.

### 2.7. Statistical Analysis

All statistical analyses were performed using SPSS27. All data are expressed as the mean ± standard deviation or median (interquartile range). Analyses were performed using the Kruskal–Wallis variance of analysis test followed by Dunn’s multiple comparison test with the statistical significance set at *p* < 0.05.

## 3. Results

### 3.1. Antioxidant Enzyme Activities

**Figure 1:**
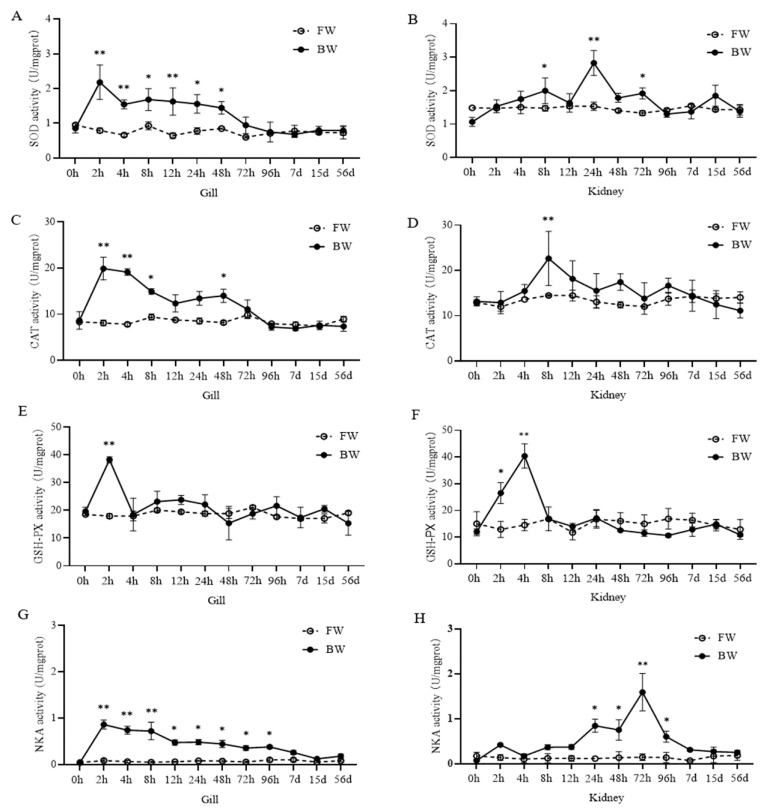
Effects of different salinities on activity of antioxidant enzymes in gill and kidney of silver carp. SOD activity in (**A**) gill and (**B**) kidney; CAT activity in (**C**) gill and (**D**) kidney; GSH-PX activity in (**E**) gill and (**F**) kidney; NKA activity in (**G**) gill and (**H**) kidney. Bars ± standard deviation (SD) with different letters are significantly different. * and ** indicate significance level of *p* < 0.05 and *p* < 0.01, respectively.

A significant increase in SOD activity was observed in the gill of silver carp exposed to brackish water between 2 and 48 h (*p* < 0.05). There was also significantly higher CAT activity in the gill of silver carp at 6 ppt salinity at 2, 4, 8, and 48 h (*p* < 0.05). GSH-PX enzyme activity in gill tissue was higher than the control group at 2 h but not significantly different (*p* > 0.05). In kidney tissues, levels of antioxidant enzymes (SOD, CAT, GSH-PX) did not significantly differ from those of the control group (*p* > 0.05). A significant difference was found between the treatment groups and the control groups when it came to NKA activity in the gill and kidney tissues (*p* < 0.05). In silver carp gill tissue, the NKA activity increased sharply in the salinity treatment group from 2 to 96 h. The NKA enzyme activity in kidney tissue increased with time from 24 to 96 h in the treatment group, peaked at 72 h, decreased gradually from 72 h to 96 d, and then finally remained stable (Figure 1).

### 3.2. Histology Structure

**Figure 2:**
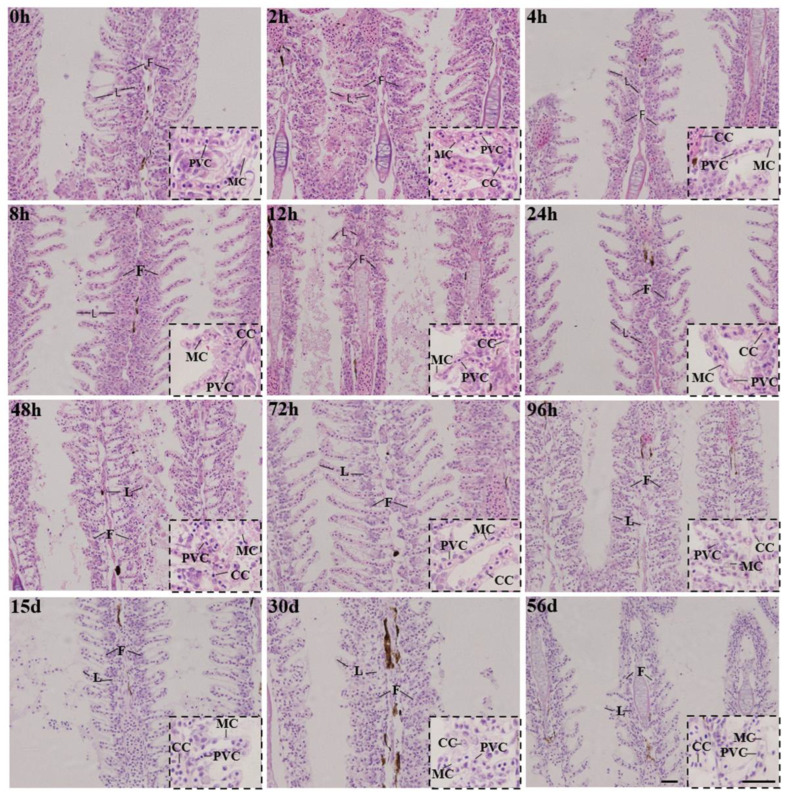
Histological structure of gill tissue of silver carps exposed at 6 ppt salinity. F: gill filament, L: gill lamellae, CC: chlorine cell, PVC: pavement cell, MC: mucous cell.

The structure of the gills changed under 6 ppt salinity exposure (Figure 2). After exposure for 2 h, the main parts of the gill filaments began to shrink, and cracks appeared. The spacing between small gills began to narrow and bend, and mucus cells became more uniformly distributed on both sides of the gill filament, flat cells became long and square, and a significant number of urinary chloride cells developed in the cracks of the gill filament stem. At 72 h, the cracks in the backbone of the gill filament had gradually grown and burst, the spacing between the gill segments gradually recovered and expanded, the increase in mucus cells peaked, urinary chloride cells appeared at the base of the gill segment, and chlorine cells in the gill filament backbone were free between the gill filaments due to the rupture of the gill filament. During 72 h~15 d, the gill filament trunk and gill patch spacing recovered, and the number of mucus and urinary chloride cells decreased. Between 15 and 56 d, the gill filament state remained basically the same, the cracking of gill filament trunk and changes to gill small sheet curvature were irreversible and evenly distributed on both sides of the gill filament, and the mucus and urinary chloride cells remained in a stable state.

### 3.3. Transcriptome Sequencing

#### 3.3.1. Quality Assessment of Transcriptome Sequencing Data

We generated 84.13 Gb of raw bases from 12 cDNA libraries. We collected 561, 014, and 006 raw reads (84.13 Gb), of which 437, 430, and 924 passed quality inspection. Clean readings per library varied in size from 5.50 to 7.75 Gb. Q20: 99.98~99.99%, Q30: 97.45~98.10%, and GC content: 45~47% were the parameters of clean readings in the FW group and BW group (Table 1). A comparison was made between the clean readings and the reference genome.

#### 3.3.2. Functional Annotation of Differentially Expressed Genes (DEGs)

**Figure 3:**
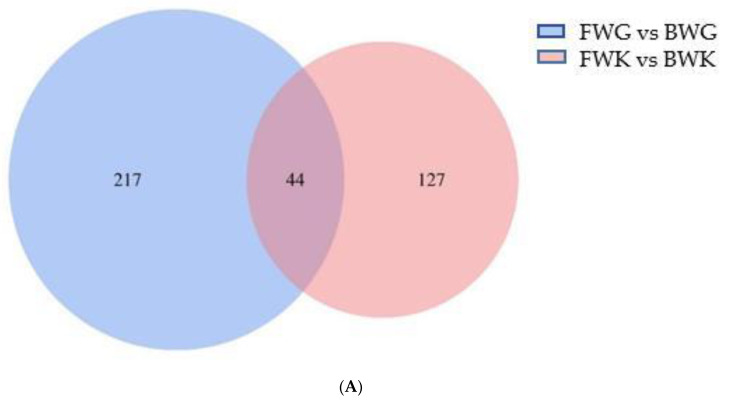

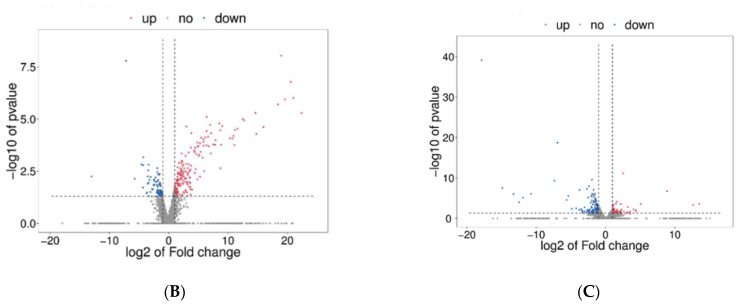
Statistics of DEG in FWG vs. BWG, FWK vs. BWK groups. (**A**) Venn diagram of DEGs in FWG vs. BWG group, FWK vs. BWK group, (**B**) upregulated and downregulated DEGs in FWG vs. BWG group, (**C**) upregulated and downregulated DEGs in FWK vs. BWK group.

P-adjust < 0.05 and log2(foldchange) ≥ 1 were used to identify DEGs with significant differences. FWK groups versus BWK groups had 432 DEGs between them compared to FWG vs. BWG (Figure 3A), including 171DEGs in gill (57 upregulated and 114 downregulated) and 261 DEGs in kidney (178 upregulated and 83 downregulated) (Figure 3B).

#### 3.3.3. GO Function Enrichment Analysis of DEGs

**Figure 4:**
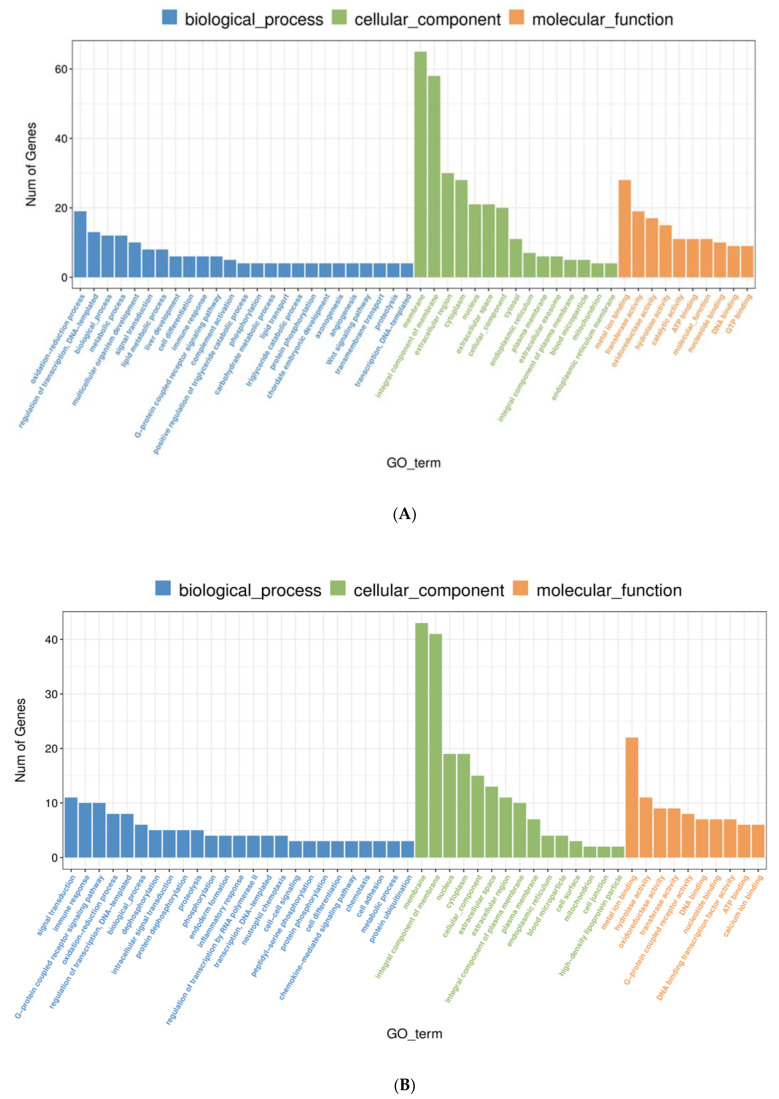
GO function enrichment analysis of DEGs in FWG vs. BWG, FWK vs. BWK groups. Top 20 enriched GO terms (*p* ≤ 0.05) in categories of biological process, cellular component, and molecular function are presented for (**A**) FWG vs. BWG and (**B**) FWK vs. BWK.

For GO functional enrichment analysis, all DEGs in the freshwater and brackish water groups were classified according to biological processes (BP), cellular component (CC), and molecular function (MF). Of the 4568 DEGs in FWG versus BWG, 1027 were enriched compared with the freshwater (control) group in 502 GO secondary entries, including 275 in biological process, 49 in cellular component, and 178 in molecular function (Figure 4A). The enriched GO terms are primarily related to immune response (GO: 0006955), and endoderm formation (GO: 0001706), G-protein coupled receptor signaling pathway (GO: 0007186), blood microparticle (GO: 0072562), extracellular space (GO: 0005615), activities of the MAP kinase tyrosine/serine/threonine phosphatases (GO: 0017017), the poly(A)-specific ribonuclease pathway (GO: 0004535), and protein tyrosine/serine/threonine phosphatase activity (GO: 0008138) are also involved. The 4568 DEGs in FWK vs. BWG 1477 were enriched in 649 GO secondary entries, including 366 in the category of biological process, 63 in cellular component, and 220 in molecular function (Figure 4B). The majority included positive regulation of triglyceride catabolic process (GO: 0010898), complement activation (GO: 0006956), phospholipid efflux (phospholipid efflux), extracellular region (GO:0005576), blood microparticle (GO: 0072562), extracellular exosome (GO: 0070062), endopeptidase inhibitor activity (GO: 0004866), and cholesterol binding (GO: 0015485) in addition to other pathways.

#### 3.3.4. KEGG Pathway Enrichment Analysis of DEGs

**Figure 5:**
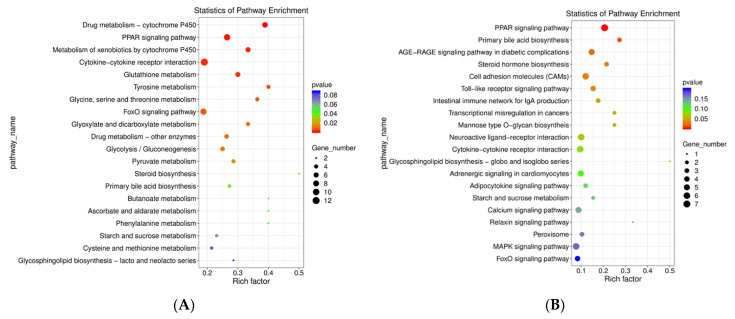
Analysis of KEGG pathway enrichment of DEGs in FWG vs. BWG; FWK vs. BWK. Top 20 KEGG pathways (*p* ≤ 0.05) for (**A**) FWG vs. BWG group; (**B**) FWK vs. BWK group (enrichment factor equals the ratio between the DEGs and all annotated genes enriched in the pathway). Each bubble’s color and size represents the significance of enrichment and the number of DEGs enriched in a pathway, respectively.

**Figure 6:**
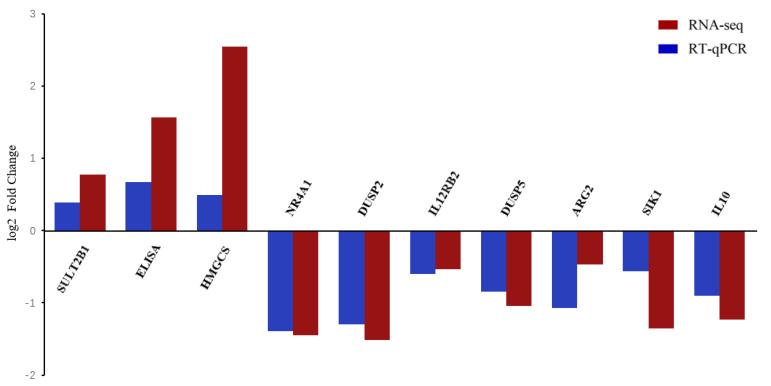
Gene expression analysis using qRT-PCR and RNA-seq for comparison. The *X*-axis shows the gene names, and the *Y*-axis shows the log2 values of the relative gene expression between the two groups. Log2 values of relative gene expression between the two groups were calculated using qRT-PCR (blue column) and RNA-seq (red column), respectively.

Significant enrichment of 162 DEGs in the KEGG pathway was observed between FWG and BWG, and 289 DEGs between FWK and BWK (Figure 5A). Between the FWG and BWG groups, there were 71 enriched KEGG pathways represented by the DEGs (Figure 5B), namely for primary bile acid biosynthesis (ko00120), PPAR signaling (ko03320), AGE-RAGE signaling associated with diabetic complications (ko04933), steroid hormone biosynthesis (ko00140), cell adhesion molecules (ko04514, CAMs), Toll-like receptor signaling (ko04620), intestinal immune network for IgA production (ko04672), transcriptional misregulation in cancers (ko05202), and mannose type O-glycan biosynthesis (ko00515), among others. Based on the 113 KEGG pathways enriched for FWK versus BWK, there was a significant enrichment of DEGs for drug metabolism—cytochrome (ko00982), PPAR signaling pathway (ko04068), metabolism of xenobiotics by cytochrome P450 (ko00980), cytokine–cytokine receptor interaction (ko04060), glutathione metabolism (ko00480), tyrosine metabolism (ko00260), glycine, serine, and threonine metabolism (ko00260), FoxO signaling (ko03320), glyoxylate and dicarboxylate metabolism (ko00630), and drug metabolism—other enzymes (ko00983), among others. In order to validate our Illumina sequencing results, we selected three upregulated (SULT2B1, ELISA, and HMGCS) and seven downregulated (NR4A1, DUSP2, IL12RB2, DUSP5, ARG2, SIK1, and IL10) DEGs for verification by qRT-PCR. The DEG expression results were obtained using qRT-PCR with designed primers (Table 2). The results showed that the expression trends of all ten DEGs were consistent with the high-throughput RNA-seq results, providing evidence of the reliability of the sequencing results (Figure 6).

## 4. Discussion

Due to the salinification of freshwater, many freshwater species might come under severe stress and threat due to their inability to cope with extreme physiological and osmotic stress. When the salinity changes, the metabolic activity, osmolarity regulation, nutritional requirements, tissue structure, and physiological and biochemical indicators are all affected in the fish. The survival rates of freshwater fish vary greatly depending on salinity conditions, even though some freshwater fish have been successfully cultivated in saline–alkali water. It is not completely understood how freshwater fish respond to varying degrees of saline–alkali stress by either strengthening or suppressing their immune systems. In this study, the differences of enzyme activity index, histology, and transcriptomics between gill and kidney tissues of juvenile silver carp were studied under BW (6‰ salinity).

### 4.1. Antioxidant Enzyme Activity

The removal of oxidative stress by antioxidant enzymes in the fish antioxidant system is remarkable: superoxide dismutase (SOD) is capable of mitigating oxidative stress. Cells are shielded from damage by the superoxide anion radical (O_2_), and H_2_O_2_ is converted to water by catalase (CAT) to maintain the stability of the intracellular environment [35]. SOD dismutase first reacts with oxygen radicals and then with oxygen. The strongest reaction is to chemical stress. When the equilibrium between reactive oxygen species (ROS) and antioxidant capacity is upset, it results in oxidative stress, which has some detrimental effects on animals [36,37]. Antioxidant enzymes play the most important role in cellular protection against oxidative stress, with the SOD–CAT–GSH-PX enzyme cascade serving as the first line of defense against ROS [38,39]. Salinity increases have previously been shown to cause behavioral changes, mortality, and induction of antioxidant enzymes in a variety of euryhaline freshwater fish species [40,41,42,43]. In the present study, salinity stress significantly increased the activity of antioxidant enzymes (SOD, CAT) in the gill tissues of silver carp. This is in accordance with the results of a previous study [35]. It also shows that increases in SOD and CAT activity stand as an indicator of surplus ROS [44,45]. In our research comparing the antioxidant enzyme activity (SOD, CAT) between gills and controls, we found that gills had the highest levels of SOD and CAT enzyme activity at 2 h. The SOD and CAT of kidney tissue reached the highest at 24 and 8 h, respectively. The results show that the range of salt tolerance is tissue specific [46]. GSH-PX enzyme activity in gill tissue was higher than the control group at 2 h but not significantly different (*p* > 0.05). In kidney tissues, although the activity of GSH-PX was significantly higher at 2 and 4 h, it was not significantly different from the control group (*p* > 0.05), which may be because gills are directly connected to the water environment and therefore respond more sensitively than the kidneys [21]. In the present study, salinity resulted in significant effects on the Na+/K+-ATPase activity of silver carp in the gill and kidney. In aquatic species, Na+/K+-ATPase is a crucial membrane protein that drives ion control and mediates whole-body osmoregulation. Therefore, Na+/K+-ATPase activities are greatly influenced by salinity [47,48]. Na+/K+ pump is mainly composed of Na+/K+-ATP enzymes, which are involved in the active transport of Na+ and K+ inside and outside cells. In studying fish salinity adaptations, the activity change of NKA enzymes and the dynamic balance of osmotic pressure are used as evaluation indicators. Whereas Na+/K+-ATPase is typically involved in the uptake of Na+ and Cl− in fresh water, it may be involved in the secretion of excesses of Na+ and Cl− in hypertonic water [49]. In the present study, we found that salinity significantly affects Na+/K+-ATPase activity of silver carp in the gill and kidney. The patterns of changes in Na+/K+-ATPase activities in the gills of freshwater fish with increasing salinity remain to be explained.

### 4.2. Histological Pathology

One of the key organs in fish that controls the osmotic pressure balance and is essential for coping with stress from the environment is the gill. While the gills of various fish have distinct anatomical differences, salinity changes have led to corresponding adaptive changes since mineralization, which was discovered in research on the effects of differing levels of salinity in causing changes in the gill tissue in rainbow trout (*Salmo irideus*) [50] and grass carp (*Ctenopharyngodon idellus*) [51]. In this study, the changes in gill filaments could be roughly divided into different periods of stress, stress recovery, recovery, and stability, during which cracks were observed in all experimental silver carp larvae for which recovery was impossible. Chloride cells are distributed in the gill filament epithelium, with the function of absorbing ions and excluding salt, and are the main cells for fish to maintain ion balance in vivo. Generally speaking, urinary chloride cells will appear in the gill base and gill filament epithelium, such as *Nile tilapia* chloride cells in which freshwater and salinity seawater are distributed in the base of the gill [52]. In addition, fewer chlorine cells are also found in the gills of freshwater sturgeon (*Acipenser schrenckii*), mainly distributed in the gill and its base, and the chlorine cell surface roof recess is small and manifests with typical freshwater chloride cell characteristics [53]. The osmotic pressure of plasma rises as the salinity of the waterbody rises. The gill tissue will produce energy to meet the energy demand for osmotic regulation. The structure then undergoes adaptive changes; however, when the salinity exceeds the fish’s tolerance limit, the gill filament tissue suffers irreversible damage, and the gill filament falls off to varying degrees, interfering with the normal physiological function of the gill tissue and resulting in fish death.

### 4.3. Transcriptome Analysis

Multiple enzymes and transporters contribute to salinity adaptation and osmotic control in surroundings with varying salinity, allowing the body to maintain its ion balance and cell permeability [54,55]. It is essential to identify genes involved in salinity changes in order to elucidate the molecular basis and factors behind this physiological process. Physiological conditions change with changes in the transcriptome, which is a series of dynamically expressed genes, so transcriptional analysis serves as an effective tool for explaining functional genomics elements and revealing the mechanisms of cell and tissue physiology [56]. This experiment conducted high-throughput sequencing of gill renal tissue transcriptome juvenile silver carp with the generation of a 137.86 Gb of raw data (5.5~8.15 Gb). After comparison annotation, 24, 1, and 166 of 24,571 transcripts were annotated using GO and 8712 genes using KEGG, which can fully explain the function of genes expressed in this sequencing. There were 171 (57 upregulated, 114 downregulated) DEGs in the FWG vs. BWG and 261 (178 upregulated, 83 downregulated) in the FWK vs. BWK. A significant number of salinity-dependent regulatory genes, in turn, verified the importance of branch filament and kidneys in osmotic pressure regulation of silver carp through the pathway enrichment of GO and KEGG of gills and kidneys in characterizing the influence of salinity on silver carp.

As salinity rises, freshwater teleosts have to contribute a significant amount of energy to adapt to higher salinity conditions and maintain their homeostatic balance [57]. It is common for freshwater fish to experience alterations in the electron transport chain, glycolysis, and fatty acid metabolism when salinity levels increase [58,59]. Pyruvate kinase, or PKM, is an enzyme that catalyzes the dephosphorylation of phosphoenolpyruvate to pyruvate, converting it into ATP and regulating glycolysis and cell metabolism [60]. Neurotransmitter-gated chloride channels and neurotransmitter membrane receptors can be activated by free amino acids, including glycine, taurine, and alanine (Glra4a, CHRNA9, CLCA4) [61]. Furthermore, in eukaryotes, lysine is acetylated to produce acetyl-CoA [62,63]. Osmoregulation involves the intermediate metabolite acetyl-CoA, which is generated through the metabolism of amino acids, lipids, and carbohydrates [64]. In our study, salt stress greatly upregulates the expression of 3-hydroxyacyl-CoA dehydrogenase genes, which may lead to the synthesis of acetyl-CoA. As a consequence, more acetyl-CoA enters the citrate cycle for energy synthesis. Protein transport and turnover are aided by ubiquitin-mediated proteolysis, ubiquinone production, and other terpenoid-quinone processes [65]. Similar results have been found in *Oreochromis niloticus* [66], *Litopenaeus vannamei* [67], and *Apostichopus japonicus* [68]. Changes in salinity cause stress, which leads to a rise in reactive oxygen species (ROS), which can cause oxidative damage [69]. The glutathione metabolic pathway plays an important role in protecting cells from ROS damage through the cell detoxification mechanism [70]. Accordingly, it was not surprising to see a marked enrichment of glutathione metabolism under salinity stress. This result is consistent with those for *Nibea albiflora* [71].

The salinity stress response mechanism of fish involves numerous stress signal transduction pathways working synergistically [71]. Cell signal transduction is the stimulation and acceptance of the stimulation process, cell membrane, or cell receptor as mediated, which impacts the biological function of the cell. The main pathways include ligands, receptors, and transduction molecules. Salinity tolerance has been identified as a function of the 14-3-3 protein family, whose members participate in a range of cellular processes, such as signal transduction, cell cycle control, apoptosis, stress responses, and malignant transformation [72,73,74]. In our study, 14-3-3 protein was upregulated in both the gill and kidney tissues. After transfer from seawater to freshwater, 14-3-3 mRNA was found to be expressed more in the gills of *F. heteroclitus* under euryhaline conditions [75,76]. Mitogen-activated protein kinases (MAPKs) are members of a family of proteins that have been shown to be involved in the conversion of osmotic sensory signals, a cellular control pathway that plays an important role in cellular responses in yeast, plants, and animals [77]. A variety of MAPK transcripts were found to be up- and downregulated in the kidney and gills of silver carp here. It has been shown that MAPK activation is associated with the small GTPases of the Rho family and in osmotic fluctuations, and they regulate actin cytoskeleton and cellular morphology [78,79]. In our study, the Rho GTPase gene showed differences in expression patterns in the different salinity groups. Accordingly, Rho GTPase is suspected to function as a cellular regulator responsible for triggering extensive cytoskeletal rearrangements [80].

Moreover, in the present study, we also found DEGs associated with immunoinflammatory response, osmoregulation, and detoxification. A previous study found that salinity stress can cause the immune systems of fish to become less effective, which can lead to a drop in immune function [81]. In general, when salinity changes in the environment where a fish is located, chaotic penetration pressure regulation can lead to reduced blood penetrant pressure and ion content and then directly to stress reactions [82], and the connection between stress and the immune system is particularly evident in lower vertebrates [83]. Interleukin-10 (IL-10) is an anti-inflammatory cytokine that regulates and inhibits the synthesis of pro-inflammatory cytokines, aiding in the natural resolution of infection and minimizing tissue damage caused by inflammation. Ion transporters and plasma membrane permeability are critical components of the osmotic control system of euryhaline fish [84,85]. The aim of this study was to examine several traditional ion transport channels and transporters. It has been shown that the solute carrier family (SLC) helps fish regulate their osmotic balance by transporting ions, proteins, and amino acids [59,86]. As a result of low-salinity stress, we observed increased expression of SLC35A4 and SLC39A7 genes. When environmental conditions (such as salinity changes) change, xenobiotics could be removed from an organism through biotransformation processes. It is possible to categorize these reactions into phase I and II reactions, depending on the pathways by which they are mediated. As an oxidative enzyme, cytochrome P450 1A (CYP1A) plays an important role in phase I biotransformations, and acclimatization of freshwater fish to high salinity may be facilitated by this method.

## 5. Conclusions

In conclusion, when the juvenile silver carp is in a brackish water environment for increasing periods of time, there will be a significant adverse effect on the antioxidant enzyme activity, tissue morphology, and osmotic pressure regulation of juvenile silver carp. As an organ directly connected with the water environment, the gill is more sensitive than the kidney. Therefore, when the salinity is 6‰, antioxidant enzyme activity causes more significant changes in gill tissue. With the increase of time, salinity will affect the morphological changes of gill filaments. The expression of significantly different genes may be related to gill remodeling and different permeability during salinity changes. On the basis of their gene ontology assignments, a number of transcripts implicated in salt tolerance were classified into four distinct functional groups. These are metabolism, signal transduction, immunoinflammatory response, and ion transportation, suggesting the processes of regulation in juvenile silver carp under brackish water conditions are complex and multifaceted. Through these mechanisms and processes, the regulatory mechanism of silver carp osmolarity from macroscopic to microscopic is revealed, which provides a theoretical basis for the study of silver carp growth in low-salinity waterbodies.

## Figures and Tables

**Table 1:** Overview of sequencing results of freshwater and brackish water groups.

Sample	Raw Data		Valid Data		Valid Ratio (Reads)	Q20%	Q30%	GC Content%
	Read	Base	Read	Base				
FW_1K	45,347,198	6.80 G	43,643,336	6.55 G	96.24	99.98	97.53	45
FW_2K	47,957,634	7.19 G	46,287,490	6.94 G	96.52	99.98	97.64	48
FW_3K	44,558,076	6.68 G	42,830,398	6.42 G	96.12	99.99	97.55	45
FW_1G	46,015,408	6.90 G	44,324,996	6.65 G	96.33	99.98	98.10	46
FW_2G	51,657,304	7.75 G	50,051,380	7.51 G	96.89	99.99	97.83	47
FW_3G	50,027,992	7.50 G	48,022,254	7.20 G	95.99	99.99	97.85	45.50
BW_1K	46,640,790	7.00 G	45,595,748	6.84 G	97.76	99.99	97.94	47
BW_2K	36,694,278	5.50 G	35,067,796	5.26 G	95.57	99.98	97.60	45
BW_3K	51,008,526	7.65 G	49,212,978	7.38 G	96.48	99.98	97.77	46
BW_1G	42,950,828	6.44 G	41,499,428	6.22 G	96.62	99.98	97.37	46
BW_2G	51,213,734	7.68 G	49,349,280	7.40 G	96.36	99.98	97.45	46
BW_3G	46,942,238	7.04 G	45,502,674	6.83 G	96.93	99.98	97.50	47

**Table 2:** Primer sequences for qRT-PCR verification.

Primer	Forward Primer Sequence (5′-3′)	Reverse Primer Sequence (5′-3′)
(Gene Name)		
SULT2B1	CAAATATACGGCTCCCTGTTG	GTGAATGAGCGAGCGATAG
ELISA	TCCGAACGGATTCCTTCTCT	ACCACTCGGTCCACTAAC
HMGCS	AGGAGGATTAGGTCATCCG	CTTCCAGACAGGCTTGTAG
NR4A1	TGTTCGTCTGACGTTCTTAATG	AGTTCTGTGAAGTGATTTCGG
DUSP2	TCATCACTGCCCTTCAGTTAGA	TGCAAAGAACCCGTCAAATC
IL12RB2	TCGTGTCACTCAATGTCTCTAA	TGAGCAGTTCACATGACTGAT
DUSP5	TACCTCCTTCCTAACACCAATG	TTTAAGGCAGCGCAGTTAT
ARG2	TGTTGCCTTCCCGAAGAT	TTGGAATGCAAAGTGGTTCA
SIK1	TCAGAGTGCCCTTCTTCAT	GAGCCACACTGAATCTCTTG
IL10	CATGATGACGTGAGTCAAGTT	GATGAAGTCCATTTGTGCCT
β-Actin	CTTTTCCAGCCATCCTTCCT	GGTCAGCAATGCCAGGGTA

## Data Availability

The datasets generated during and/or analyzed during the current study are available from the corresponding author on reasonable request.
